# Adherence to the Mediterranean Diet and Mortality after Breast Cancer

**DOI:** 10.3390/nu12123649

**Published:** 2020-11-27

**Authors:** Matteo Di Maso, Luigino Dal Maso, Livia S. A. Augustin, Antonella Puppo, Fabio Falcini, Carmen Stocco, Veronica Mattioli, Diego Serraino, Jerry Polesel

**Affiliations:** 1 Department of Clinical Sciences and Community Health, Branch of Medical Statistics, Biometry and Epidemiology G.A. Maccacaro, Università degli Studi di Milano, via A. Vanzetti 5, 20133 Milan, Italy; matteo.dimaso@unimi.it; 2 Department of Public Health and Pediatric Sciences, Università degli Studi di Torino, CTO Hospital, via G. Zuretti 29, 10126 Turin, Italy; 3 Unit of Cancer Epidemiology, Centro di Riferimento Oncologico di Aviano IRCCS, via Gallini 2, 33081 Aviano, Italy; dalmaso@cro.it (L.D.M.); vmattioli@cro.it (V.M.); serrainod@cro.it (D.S.); 4 Unit of Epidemiology, Istituto Tumori Fondazione Pascale, IRCSS, via M. Semmola 1, 80131 Naples, Italy; l.augustin@istitutotumori.na.it; 5 IRCCS Ospedale Policlinico San Martino, Liguria Cancer Registry, Largo R. Benzi 10, 16132 Genova, Italy; antonella.puppo@hsanmartino.it; 6 Romagna Cancer Registry, Istituto Scientifico Romagnolo per lo Studio e la Cura dei Tumori (IRST), IRCCS, via P. Maroncelli 40, 47014 Meldola, Italy; fabio.falcini@irst.emr.it; 7 Azienda Usl Della Romagna, via della Rocca 19, 47121 Forlì, Italy; 8 Veneto Tumour Registry, Azienda Zero, via J. Avanzo 35, 35132 Padova, Italy; carmen.stocco@azero.veneto.it

**Keywords:** Mediterranean diet, breast cancer, mortality, survival

## Abstract

Adherence to Mediterranean diet has been consistently associated with a reduced mortality in the general population, but evidence for women with breast cancer is scanty. Methods: A cohort of 1453 women with breast cancer diagnosed between 1991 and 1994 in northern Italy was followed-up for vital status for 15 years after diagnosis. The pre-diagnostic habitual diet was assessed through a structured questionnaire and adherence to the Mediterranean diet was evaluated through the Mediterranean Diet Score. Hazard ratios (HR) of death with confidence intervals (CI) were estimated using Cox model, adjusting for potential confounders. Results: Compared to women who scarcely adhere to the Mediterranean diet (*n* = 332, 22.8%), those highly adherent (*n* = 500, 34.4%) reported higher intakes of carbohydrates, mono-unsaturated and poly-unsaturated fatty acids, vitamins, folate, and carotenoids, and lower intakes of cholesterol and animal proteins. Adherence to the Mediterranean diet was associated with a better prognosis: 15-year overall survival of 63.1% for high and 53.6% for low adherence, respectively (*p* = 0.013). HR for all-cause mortality was 0.72 (95% CI: 0.57−0.92) and HR for breast cancer mortality was 0.65 (95% CI: 0.43−0.98) for women 55 years and older. No significant association emerged for breast cancer mortality in the total cohort. Conclusions: Although dietary habits may have changed after breast cancer diagnosis, these findings indicate that women who ate according to the Mediterranean dietary pattern prior to their diagnosis may have greater chance of a favorable prognosis after breast cancer diagnosis compared to those who did not.

## 1. Introduction

Breast cancer (BrCa) is, by far, the most common cancer among women worldwide accounting for over two million cases each year [1]. In Europe, although BrCa still counts as the leading cause of cancer death among women [1], the 5-year survival is above 80% [2]. As a consequence, a considerable number of women are living after a diagnosis of BrCa. For instance, 2.6% of all Italian women (approximately 0.8 million persons) will be alive in 2020 after a breast cancer diagnosis [3]. The identification of modifiable factors affecting mortality after BrCa is therefore of great interest.

The association between diet and BrCa has been extensively investigated considering food groups, single foods, and nutrients [4], including their impact on survival. Favorable oncologic outcomes have been associated to high intakes of fibers [5,6], β-carotene [5,7], and proteins [8], and to low intake of fats [5,9]. However, findings on individual foods and nutrients provided limited information on the impact of the overall diet on cancer survival, since they do not fully consider the interaction between nutrients. To overcome this problem, dietary patterns have been proposed as a combination of the specific nutritional indicators to evaluate the adherence to healthy diets; these studies reported reduced mortality in BrCa patients following healthy diets (defined according to healthy eating indexes) [10,11], in contrast to Western diet [10].

The Mediterranean diet is unanimously considered a healthy dietary pattern, characterized by high intakes of fruits, vegetables, whole grain products, pulses and fish, and by limited intakes of dairy products and red meat [12]. Adherence to the Mediterranean diet has been consistently associated with a reduced all-cause mortality in the general population, with similar associations across geographic areas [13]; it is therefore of interest to evaluate whether women with BrCa eating according to the Mediterranean diet may benefit from a similar effect on survival. Lower all-cause mortality, but not a cancer-specific one, has been reported among cancer patients adherent to the Mediterranean diet [14]. However, evidence for BrCa is scanty, with only one analysis of the Nurses’ Health study [15] suggesting a reduction of approximately 15% in all-cause mortality, and 20% in non-BrCa mortality in BrCa patients highly adherent to the Mediterranean diet, compared to those scarcely adherent.

Therefore, the present study aims at investigating the association between adherence to the Mediterranean diet at diagnosis and long-term mortality in a cohort of women with breast cancer living in Italy, an area where the Mediterranean diet is widespread.

## 2. Materials and Methods

This study analyzed data from a retrospective cohort of women with BrCa initially enrolled as cases in an Italian case-control study on the association between lifestyle factors and BrCa risk, whose study design was previously described [16,17]. Study protocol was approved by the Board of Ethics of the participating centers. All patients signed an informed consent at enrollment. Cohort participants were 1453 consecutive patients aged 23−78 years (median age: 55 years) with incident, histologically confirmed BrCa diagnosed during the period 1991−1994 in the major hospitals of the provinces of Pordenone and Forlì and the urban area of Genoa in northern Italy. None of them had any prior cancer diagnosis or received previous cancer treatment.

Clinical characteristics at diagnosis, including tumor TNM stage and estrogen/progesterone receptor (ER/PR) status, were gathered from medical records and were centrally reviewed by a clinician. During hospitalization, BrCa patients were interviewed by trained personnel using a structured questionnaire including information on age, education, socio-demographic characteristics, anthropometric measures, lifestyle habits (e.g., drinking and smoking habits, physical activity), personal medical history, and family history of cancer. Body mass index (BMI) was computed as weight (kg) divided by height squared (m^2^).

The habitual diet during the two years prior to cancer diagnosis was assessed through a validated [18] and reproducible [19] food-frequency questionnaire (FFQ). The FFQ included 78 foods, beverages or recipes (i.e., the most common ones in the Italian diet) structured into 7 sections: (i) cereals, bread, and first courses; (ii) second courses (i.e., meat, fish, and other main dishes); (iii) side dishes (i.e., vegetables); (iv) fruits; (v) desserts, sweets, and soft drinks; (vi) hot beverages, milk, and sweeteners; (vii) alcoholic beverages. Patients were asked to indicate the average weekly frequency of consumption of each dietary item. Intakes lower than once a week but at least once a month were coded as 0.5 per week. Seasonal consumption was elicited for fruit and vegetables subject to seasonal variation. Daily intakes of food groups were derived from the FFQ by weighting the daily consumption of relevant food items for the corresponding serving weight and summing them up to the food group total. They were expressed in grams per day (g/day). Total energy and nutrient intake were computed using an Italian food composition database [20].

Adherence to Mediterranean diet was investigated using the Mediterranean Diet Score (MDS). This is an a priori score developed using 9 dietary indicators [21], which were high consumption of: cereals, fruit, vegetables, legumes, fish; high monounsaturated/saturated fatty acids (MUFA/SFA) ratio; low consumption of: dairy products (including milk) and meat; and moderate alcohol consumption. For each food group and nutrient, high or low consumption was defined according to its median value; for alcohol intake, moderate alcohol consumption was defined as 1−3 drinks/day. For each study participant and each diet indicator, a value of 1 was assigned when the subject fulfilled the MDS requirement, 0 otherwise. The MDS was calculated adding up the values for each of the 9 components; thus, the score ranged from 0 (representing minimal adherence) to 9 (maximal adherence). According to MDS, adherence to Mediterranean diet was classified as low (MDS: 0−3), moderate (MDS: 4−5), or high (MDS: 6−9).

The vital status, the date of death, and the underlying cause of death were ascertained through a record-linkage procedure with the population-based regional cancer registries covering the areas where patients were enrolled [17]. Person-time at risk was computed from the date of BrCa diagnosis to the date of death or to the end of follow-up, whichever came first. Median length of follow-up was 12.6 years (maximum: 16.8 years). The follow-up for the present analysis was truncated at 15 years after diagnosis.

The overall survival probabilities according to MDS were estimated by means of the Kaplan–Meier method and survival differences were tested through the log-rank test [22]. To account for competing risks, cause-specific mortality was evaluated through cumulative incidence [23], and differences according to MDS were tested through Gray’s test [24]. Hazard ratios (HRs) of death for all-causes and corresponding 95% confidence intervals (CIs) were estimated using Cox Proportional Hazards model [22], after checking for the proportional hazards assumption through the inspection of Schoenfeld residuals and including interactions with follow-up time [22]. HRs were adjusted for study design variables (area of residence, calendar period of cancer diagnosis), socio-demographic characteristics (age at diagnosis, education, menopausal status), clinical cancer features (TNM stage, ER/PR status), and total energy intake. Kruskal–Wallis test was used to test differences in food groups or nutrient intake according to levels of adherence to the Mediterranean diet. Statistical significance was claimed for *p* < 0.05 (two-tailed).

## 3. Results

At BrCa diagnosis, 500 (34.4%) patients reported high adherence to Mediterranean diet (MDS = 6−9) whereas 332 (22.8%) reported low adherence (MDS = 0−3; Table 1). Adherence to Mediterranean diet was significantly higher in patients younger than 45 years than those aged ≥45 years (*p* = 0.028), and in current drinkers than in never drinkers (*p* < 0.001; Table 1). 

Women highly adherent to the Mediterranean diet reported higher intakes of cereals, fish, fruit, vegetables and legumes, and a lower intake of dairy products (Table 2). In patients highly adherent to the Mediterranean diet, fats and proteins from vegetable sources were preferred to those from animal sources (Table 3). Further, intake of carbohydrates, mono-unsaturated fatty acids, poly-unsaturated fatty acids, vitamins, folate, and carotenoids significantly increased with increasing adherence to Mediterranean diet, whereas cholesterol intake showed an opposite trend.

**Figure 1:**
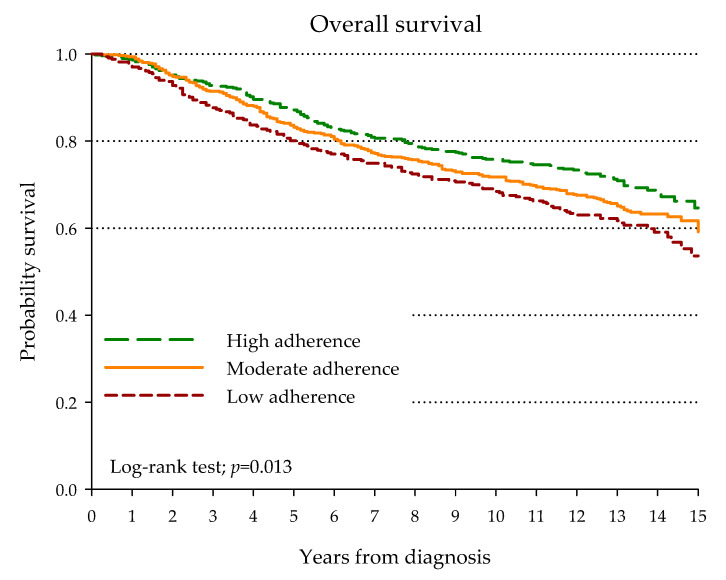
Kaplan–Meier estimates of overall survival according to adherence to the Mediterranean diet.

During the 15-year follow-up, 503 patients (34.6%) had died with a median time to death of 64 months. Overall, 365 (72.6%) were attributed to BrCa and 138 (27.4%) to other causes. BrCa patients highly adherent to Mediterranean diet reported a higher survival than those with low adherence (*p* = 0.013): the 15-year survival probability was 63.1% and 53.6% for high and low adherence, respectively (Figure 1). The advantage in overall survival in women with high MDS compared to low MDS was confirmed by multivariate analysis (Table 4), with HR for death of 0.72 (95% CI: 0.57−0.92).

**Figure 2:**
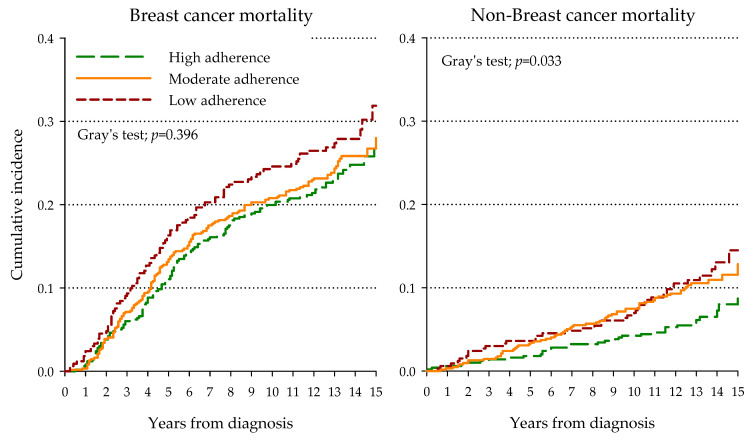
Kaplan–Meier estimates of cumulative incidence of breast cancer and non-breast cancer mortality according to adherence to Mediterranean diet.

Adherence to Mediterranean diet was not significantly associated to BrCa-specific mortality (*p* = 0.396, Figure 2), although the cumulative incidence of BrCa-related deaths was higher among women with low adherence. Non-BrCa-specific mortality was significantly lower among women highly adherent to Mediterranean diet (*p* = 0.033). These findings were confirmed by the multivariate model (Table 4), where high adherence to the Mediterranean diet was significantly associated with reduced non-BrCa-related mortality alone (HR = 0.58; 95% 0.36−0.93).

The association between Mediterranean diet and mortality was further investigated according to strata of age, menopausal status, and BMI (Table 5). No significant associations emerged in women diagnosed at age <55 years. Conversely, in those aged ≥55 years, high adherence to Mediterranean diet was significantly inversely associated with overall mortality (HR = 0.55; 95% CI: 0.39−0.76), as well as BrCa mortality (HR = 0.65; 95% CI: 0.43−0.98) and non-BrCa mortality (HR = 0.51; 95% CI: 0.30−0.90). A similar pattern emerged for menopausal status: a significant inverse association between adherence to the Mediterranean diet and mortality emerged only among postmenopausal women, though the effect on BrCa mortality in post-menopausal women was not statistically significant. Further, the inverse association between adherence to the Mediterranean diet and mortality was more evident in overweight/obese patients than in those with normal weight. Indeed, among patients with BMI ≥ 25 kg m^−2^, the risk of all-cause death was 0.70 (95% CI: 0.51−0.96) in patients moderately adherent to the Mediterranean diet and 0.64 (95% CI: 0.45−0.92) in those highly adherent. No such associations emerged in women with BMI < 25 kg m^−2^.

## 4. Discussion

The results of the present study support a beneficial role of prediagnostic Mediterranean diet in survival in women with BrCa, with reductions in the risk of all-cause and non-BrCa-associated deaths. The beneficial effect of the high adherence to the Mediterranean diet is particularly evident in women 55 years and older who also showed a reduced risk of BrCa-specific death.

Adherence to the Mediterranean diet has been associated with reduced incidence of several health outcomes [25], including overall mortality and cancer-specific mortality [13,25]. Nonetheless, very few studies have been conducted on survival in cancer patients [15,26,27]. In particular, only one previous analysis in post-menopausal women focused on breast cancer [15]: in comparison to women scarcely adherent to the Mediterranean diet, those who were highly adherent reported a 26% reduction in all-cause mortality and a 42% reduction in non-BrCa-specific mortality. However, these associations were no longer significant when lifestyle and clinical variables were included as covariates.

The favorable effect of the Mediterranean diet on mortality and other health outcomes has been usually attributed to the additive health benefits of each nutrient that characterize this diet [13]. High fiber intake has been associated with a reduction in both all-cause and BrCa-specific mortality, with a strong linear relationship [6]. Similarly, elevated dietary intake of β-carotene, a marker of vegetable and fruit intake, has been associated with a 30% decrease in overall mortality [7]. Furthermore, lower dietary fat intake, especially from animal origin, has been associated with improved survival after BrCa [5,9]. These findings are in line with the results of the present study; although we did not evaluate the association between single components of the Mediterranean diet and mortality, women highly adherent to the Mediterranean diet reported higher daily intakes of fiber, vegetable fats and carotenoids, and lower intakes of animal fats than women scarcely adherent. Several studies reported an advantage in overall survival and progression-free survival with increased protein intake [8]. In the present study, total protein intake, particularly from vegetable sources, increased with increasing adherence to the Mediterranean diet. However, it is worth noting that the increase in total protein in our study was mainly due to those from vegetable sources, whereas those from animal sources declined with increasing MDS.

In this study, adherence to the Mediterranean diet did not modify the survival in women younger than 55 years at diagnosis. Conversely, among women older than 55 years, high adherence to the Mediterranean diet reduced both BrCa and non-BrCa mortality. Similarly, no effect of Mediterranean diet emerged for pre-menopausal BrCa. Young women are affected more frequently by aggressive BrCa molecular subtypes [28], in particular by triple-negative BrCa, which is associated with poor prognosis and early death [28]. Therefore, it is likely that the prognosis of these patients is scarcely affected by lifestyle factors [9]. This could partly explain the lack of association between the Mediterranean diet and BrCa outcomes in patients younger than 55 years. Indeed, although triple-negative BrCa could not be identified in our study due to the lack of information on human epidermal growth factor receptor 2 status, the prevalence of ER^-^/PR^-^ was higher among women aged <55 years at diagnosis than among older ones (i.e., 45.8% and 37.6%, respectively).

In the present study, the beneficial effect of the Mediterranean diet on BrCa prognosis was more evident among patients with BMI ≥ 25 kg m^−2^. A worse prognosis in overweight and obese women has already been reported in the present study population [17]. It is therefore possible that the beneficial effect of the Mediterranean diet emerged more where the baseline risk of death was higher, through the reduction of other comorbidities that impact survival. Clinical and observational studies have consistently reported inverse associations between adherence to the Mediterranean diet and risk of metabolic syndrome [29]. Besides the overweight factor and obesity, the Mediterranean diet has been associated with each medical condition defining the metabolic syndrome, such as hypercholesterolemia, hypertension, hypertriglyceridemia and hyperglycemia [29]. Furthermore, epigenetic modifications due to the Mediterranean diet has recently been reported in an interventional study conducted on overweight BrCa patients [30] which showed significant changes in the expression of 42 extracellular miRNAs after an 8-week Mediterranean dietary treatment. Genetic pathways regulated by the differentially expressed miRNAs include signaling associated with BrCa, energy metabolism, glucose metabolism, and insulin [30].

The lack of information on modifications of dietary habits after BrCa diagnosis was a major limitation in our current study. Although an increase in vegetable and fruit consumption after diagnosis has been reported in French women [31], this was unlikely to have occurred in Italian women diagnosed with BrCa between 1991 and 1994 since, at that time, the general population had not been made aware of any putative association between diet and cancer survival, and no dietary guidelines existed for patients with BrCa. Furthermore, studies investigating dietary modification in relation to BrCa survival consistently reported similar results when adherence to healthy diets was measured before and after BrCa [10]. The lack of information on type of treatment after BrCa diagnosis may be another study weakness. Selection bias may also have occurred. However, study patients were women previously enrolled as cases in a hospital-based case-control study [16] recruiting all newly diagnosed BrCa patients consecutively admitted to the major local hospitals in the study areas; no selection for specific clinical characteristics or treatments was done and refusal rate and loss to follow-up was below 5%. Thus, the present study population could be considered representative of BrCa patients in the study areas.

## 5. Conclusions

The findings of the present study further support the beneficial impact of the Mediterranean on BrCa outcomes, in particular in those overweight or obese and in older women. Although results might be biased by the lack of postdiagnosis dietary information, these results indicate that women who ate according to the Mediterranean diet prior to BrCa diagnosis had a higher chance of a favorable prognosis. Thus, in agreement with current cancer prevention guidelines [32], this study encourages women to follow a healthy diet. In summary, the Mediterranean diet, which has been associated with longevity in otherwise healthy subjects may also prolong life in women with a BrCa diagnosis.

## Figures and Tables

**Table 1:** Distribution of 1453 women diagnosed with breast cancer, according to baseline socio-demographic and clinical characteristics and Mediterranean Diet Score (MDS).

	Adherence to Mediterranean Diet (MDS)						
	Low (0–3)		Moderate (4–5)		High (6–9)		
	*n*	(%)	*n*	(%)	*n*	(%)	
All patients	332	(22.8)	621	(42.7)	500	(34.4)	
Age at diagnosis (years)							
<45	53	(20.4)	109	(41.9)	98	(37.7)	
45–54	108	(25.1)	168	(39.1)	154	(35.8)	
55–64	95	(21.1)	191	(42.4)	165	(36.6)	
≥65	76	(24.4)	153	(49.0)	83	(26.6)	*p* = 0.028
Education (yrs) ^1^							
<7	186	(25.3)	318	(43.3)	230	(31.3)	
7–11	92	(22.1)	168	(40.4)	156	(37.5)	
≥12	52	(17.5)	134	(45.0)	112	(37.6)	*p* = 0.058
Menopausal status							
Pre/Peri	121	(21.8)	237	(42.9)	195	(35.3)	
Post	211	(23.4)	384	(42.7)	305	(33.9)	*p* = 0.757
Current tobacco smoking							
No	255	(21.9)	509	(43.8)	399	(34.3)	
Yes	77	(26.6)	112	(38.6)	101	(34.8)	*p* = 0.163
BMI (kg m^−2^) ^1^							
<25	178	(21.9)	342	(42.0)	296	(36.1)	
25–29.9	110	(23.7)	199	(42.9)	155	(33.4)	
≥30	44	(25.6)	78	(45.4)	50	(29.1)	*p* = 0.574
TNM stage							
I	106	(22.3)	190	(40.0)	179	(37.7)	
II	141	(22.0)	287	(44.7)	214	(33.3)	
III-IV	45	(23.3)	80	(41.5)	68	(35.2)	
Unknown	40	(28.0)	64	(44.8)	39	(27.3)	*p* = 0.284
ER/PR status							
ER^+^/PR^+^	46	(31.3)	58	(39.5)	43	(29.3)	
ER^−^/PR^+^	12	(23.1)	18	(34.6)	22	(42.3)	
ER^+^/PR^−^	20	(21.7)	39	(42.4)	33	(35.9)	
ER^−^/PR^−^	136	(22.6)	267	(44.3)	200	(33.2)	
Unknown	118	(21.1)	239	(42.8)	202	(36.1)	*p* = 0.279

BMI: body mass index; ER: estrogen receptor (^−^: Negative; ^+^: Positive); PR: progesterone receptor. ^1^ The sum does not add up to total because of missing values.

**Table 2:** Median intake of food groups and interquartile range (Q1–Q3) according to adherence to Mediterranean diet.

Food Group	Low Adherence		Moderate Adherence		High Adherence		Kruskal–Wallis Test
	Median	(Q1–Q3)	Median	(Q1–Q3)	Median	(Q1–Q3)	
Cereals (g)	152	(119–188)	172	(132–220)	200	(159–253)	*p* < 0.001
White meat (g)	57	(29–86)	57	(29–86)	57	(29–71)	*p* = 0.180
Red/processed meat (g)	96	(67–125)	96	(66–128)	91	(63–124)	*p* = 0.343
Fish (g)	21	(11–27)	27	(16–43)	33	(27–49)	*p* < 0.001
Dairy products (g)	286	(196–398)	250	(109–336)	168	(88–284)	*p* < 0.001
Fruits (g)	163	(127–200)	199	(156–264)	252	(215–300)	*p* < 0.001
Vegetables (g)	289	(189–361)	366	(241–521)	458	(343–590)	*p* < 0.001
Legumes (g)	23	(14–32)	32	(18–50)	46	(32–68)	*p* < 0.001
Desserts (g)	43	(17–78)	40	(18–74)	38	(18–71)	*p* = 0.745

**Table 3:** Nutrient median values and interquartile range (Q1–Q3) according to adherence to Mediterranean diet.

Daily Nutrient Intake	Low Adherence		Moderate Adherence		High Adherence		Kruskal–Wallis Test
	Med	(Q1–Q3)	Med	(Q1–Q3)	Med	(Q1–Q3)	
Total proteins (g)	85.5	(72.0–99.2)	87.5	(74.4–104.3)	89.9	(74.9–105.5)	*p* = 0.017
Vegetable proteins (g)	25.6	(20.6–30.2)	29.1	(24.7–34.9)	33.3	(27.8–39.4)	*p* < 0.001
Animal proteins (g)	59.8	(48.9–71.4)	58.0	(47.4–70.8)	56.2	(44.4–68.2)	*p* = 0.005
Total fats (g)	75.6	(62.5–94.6)	80.9	(65.0–100.3)	88.3	(71.1–108.6)	*p* < 0.001
Vegetable fats (g)	31.0	(23.3–41.7)	37.0	(27.9–48.6)	47.7	(35.4–60.9)	*p* < 0.001
Animal fats (g)	44.7	(34.2–55.7)	42.4	(32.9–53.5)	39.4	(30.7–50.3)	*p* < 0.001
Carbohydrates (g)	242.4	(193.9–292.3)	268.8	(225.1–322.7)	292.3	(245.4–352.0)	*p* < 0.001
Sugars (g)	90.8	(68.4–115.6)	100.7	(77.8–126.7)	107.4	(86.4–134.8)	*p* < 0.001
Starch (g)	147.8	(117.3–185.2)	161.0	(129.9–204.8)	185.6	(149.8–224.4)	*p* < 0.001
Fiber (g)	19.4	(15.5–23.3)	23.4	(19.6–28.2)	27.5	(23.5–32.5)	*p* < 0.001
SAFA (g)	27.5	(21.6–34.3)	27.7	(21.5–34.2)	27.5	(21.3–34.5)	*p* = 0.908
MUFA (g)	32.2	(24.8–40.7)	35.3	(27.4–45.0)	42.6	(32.3–54.0)	*p* < 0.001
PUFA (g)	10.9	(8.2–14.6)	11.3	(8.5–16.1)	12.1	(9.6–15.6)	*p* = 0.001
Cholesterol (mg)	316.6	(251.8–403.6)	320.1	(254.3–405.9)	304.6	(236.6–379.2)	*p* = 0.049
Calcium (g)	1.06	(0.82–1.30)	0.99	(0.77–1.29)	0.96	(0.75–1.23)	*p* = 0.033
Potassium (g)	3.37	(2.77–4.06)	3.74	(3.19–4.37)	4.07	(3.51–4.69)	*p* < 0.001
Iron (mg)	12.6	(10.3–14.8)	13.6	(11.5–16.2)	14.8	(12.6–17.6)	*p* < 0.001
Thiamin (mg)	0.80	(0.64–0.95)	0.85	(0.71–0.99)	0.91	(0.76–1.07)	*p* < 0.001
Riboflavin (mg)	1.50	(1.26–1.91)	1.52	(1.21–1.88)	1.52	(1.26–1.85)	*p* = 0.985
Vitamin C (mg)	114.9	(81.3–148.8)	142.1	(100.1–190.2)	165.8	(127.8–215.8)	*p* < 0.001
Vitamin B6 (mg)	1.70	(1.39–2.01)	1.86	(1.56–2.19)	1.97	(1.68–2.29)	*p* < 0.001
Folate (µg)	231.1	(190.9–275.4)	267.5	(218.4–311.8)	293.5	(251.3–346.7)	*p* < 0.001
Niacin (mg)	15.6	(12.7–18.2)	16.9	(14.2–19.9)	18.4	(15.5–21.1)	*p* < 0.001
α-Carotene (µg)	449.5	(185.7–786.8)	587.1	(308.1–957.6)	792.3	(479.7–1237)	*p* < 0.001
β-Carotene (mg)	3.91	(2.95–5.02)	4.88	(3.69–6.11)	5.88	(4.68–7.26)	*p* < 0.001
Vitamin D (µg)	2.43	(1.86–3.15)	2.85	(2.13–3.62)	3.23	(2.53–4.17)	*p* < 0.001
Vitamin E (mg)	11.7	(9.30–15.0)	13.9	(11.2–17.1)	16.8	(13.5–20.1)	*p* < 0.001
Total energy (kJ)	8473	(7213–10339)	9192	(7849–10841)	9937	(8372–11673)	*p* < 0.001

Med: Median value.

**Table 4:** Hazard ratios (HR) of death and corresponding 95% confidence intervals (CI) by adherence to the Mediterranean diet.

Adherence to Mediterranean Diet	Patients	Deaths		HR (95% CI) ^1^
		*n*	(%)	
**All-cause mortality**				
Low	332	132	(43.8)	Reference
Moderate	621	220	(35.4)	0.86 (0.69–1.07)
High	500	151	(30.2)	0.72 (0.57–0.92)
**Breast cancer mortality**				
Low	332	93	(28.0)	Reference
Moderate	621	154	(24.8)	0.88 (0.67–1.15)
High	500	118	(23.6)	0.83 (0.62–1.11)
**Non-breast cancer mortality**				
Low	332	41	(12.1)	Reference
Moderate	621	63	(10.3)	0.87 (0.58–1.29)
High	500	34	(6.8)	0.58 (0.36–0.93)

^1^ Estimated using Cox proportional hazard model adjusted for area of residence at diagnosis, calendar period of cancer diagnosis, age at diagnosis, years of education, menopausal status, TNM stage, estrogen/progesterone receptor status, and total energy intake. Cause-specific mortality was further adjusted for competing risk according to Fine-Gray model.

**Table 5:** Hazard ratios (HR) ^1^ of death and corresponding 95% confidence intervals (CI) by adherence to the Mediterranean diet according to selected strata.

	Age (Years)		Menopausal Status		BMI (kg m^−2^)	
	<55	≥55	Pre/Peri	Post	<25	≥25
**All-cause mortality**						
Low	Reference	Reference	Reference	Reference	Reference	Reference
Moderate	1.04 (0.72–1.51)	0.76 (0.58–1.00)	1.06 (0.69–1.62)	0.79 (0.61–1.02)	0.97 (0.71–1.33)	0.70 (0.51–0.96)
High	1.01 (0.69–1.48)	0.55 (0.39–0.76)	1.01 (0.65–1.58)	0.65 (0.48–0.87)	0.81 (0.58–1.14)	0.64 (0.45–0.92)
**Breast cancer mortality**						
Low	Reference	Reference	Reference	Reference	Reference	Reference
Moderate	1.03 (0.69–1.56)	0.78 (0.54–1.12)	1.04 (0.65–1.69)	0.80 (0.57–1.12)	1.14 (0.77–1.69)	0.65 (0.44–0.97)
High	1.06 (0.69–1.61)	0.65 (0.43–0.98)	1.06 (0.65–1.71)	0.73 (0.51–1.05)	0.97 (0.64–1.46)	0.73 (0.48–1.11)
**Non-breast cancer mortality**						
Low	Reference	Reference	Reference	Reference	Reference	Reference
Moderate	0.88 (0.34–2.33)	0.86 (0.55–1.34)	0.84 (0.28–2.51)	0.88 (0.55–1.31)	0.68 (0.39–1.16)	0.99 (0.54–1.81)
High	0.67 (0.27–1.82)	0.51 (0.30–0.90)	0.67 (0.24–1.87)	0.58 (0.34–0.99)	0.62 (0.33–1.15)	0.51 (0.24–1.08)

^1^ Estimated using Cox Proportional Hazard Model adjusted for area of residence at diagnosis, calendar period of cancer diagnosis, age at diagnosis, years of education, TNM stage, estrogen/progesterone receptor status, and total energy intake, when appropriate.

## References

[1] Ferlay J., Colombet M., Soerjomataram I., Mathers C., Parkin D.M., Piñeros M., Znaor A., Bray F. (2019). Estimating the global cancer incidence and mortality in 2018: GLOBOCAN sources and methods. Int. J. Cancer.

[2] Sant M., Chirlaque Lopez M.D., Agresti R., Sánchez Pérez M.J., Holleczek B., Bielska-Lasota M., Dimitrova N., Innos K., Katalinic A., Langseth H. (2015). Survival of women with cancers of breast and genital organs in Europe 1999–2007: Results from the EUROCARE-5 study. Eur. J. Cancer.

[3] Guzzinati S., Virdone S., De Angelis R., Panato C., Buzzoni C., Capocaccia R., Francisci S., Gigli A., Zorzi M., Tagliabue G. (2018). Characteristics of people living in Italy after a cancer diagnosis in 2010 and projections to 2020. BMC Cancer.

[4] De Cicco P., Catani M.V., Gasperi V., Sibilano M., Quaglietta M., Savini I. (2019). Nutrition and breast cancer: A literature review on prevention, treatment and recurrence. Nutrients.

[5] McEligot A.J., Largent J., Ziogas A., Peel D., Anton-Culver H. (2006). Dietary fat, fiber, vegetable, and micronutrients are associated with overall survival in postmenopausal women diagnosed with breast cancer. Nutr. Cancer.

[6] Jayedi A., Emadi A., Khan T.A., Abdolshahi A., Shab-Bidar S. (2020). Dietary fiber and survival in women with breast cancer: A dose-response meta-analysis of prospective cohort studies. Nutr. Cancer.

[7] He J., Gu Y., Zhang S. (2018). Vitamin A and breast cancer survival: A systemic review and meta-analysis. Clin. Breast Cancer.

[8] Holmes M.D., Wang J., Hankinson S.E., Tamimi R.M., Chen W.Y. (2017). Protein intake and breast cancer survival in the Nurses’ Health Study. J. Clin. Oncol..

[9] Chlebowski R.T., Aragaki A.K., Anderson G.L., Simon M.S., Manson J., Neuhouser M.L., Pan K., Stefanick M.L., Rohan T.E., Lane D. (2018). Association of low-fat dietary pattern with breast cancer overall survival. A secondary analysis of the Women’s Health Initiative randomized clinical trial. JAMA Oncol..

[10] Jochems S.H.J., Van Osch F.H.M., Bryan R.T., Wesselius A., van Schooten F.J., Cheng K.K., Zeegers M.P. (2017). Impact of dietary patterns and the main food groups on mortality and recurrence in cancer survivors: A systemic review of current epidemiological literature. BMI Open.

[11] Terranova C.O., Protani M.M., Reeves M.M. (2018). Overall dietary intake and prognosis after breast cancer: A systemic review. Nutr. Cancer.

[12] Mazzocchi A., Leone L., Agostoni C., Pali-Schöll I. (2019). The secrets of Mediterranean diet. Does [only] olive oil matter?. Nutrients.

[13] Eleftheriou D., Benetou V., Trichopoulou A., La Vecchia C., Bamia C. (2018). Mediterranean diet and its components in relation to all-cause mortality: Meta-analysis. Br. J. Nutr..

[14] Morze J., Danielewicz A., Przybyłowicz K., Zeng H., Hoffmann G., Schwingshackl L. (2020). An updated systemic review and meta-analysis on adherence to Mediterranean diet and risk of cancer. Eur. J. Nutr..

[15] Kim E.H.J., Willett W.C., Fung T., Rosner B., Holmes M.D. (2011). Diet quality indices and postmenopausal breast cancer survival. Nutr. Cancer.

[16] Franceschi S., Favero A., Decarli A., Negri E., La Vecchia C., Ferraroni M., Russo A., Salvini S., Amadori D., Conti E. (1996). Intake of macronutrients and risk of breast cancer. Lancet.

[17] Dal Maso L., Zucchetto A., Talamini R., Serraino D., Stocco C.F., Vercelli M., Falcini F., Franceschi S. (2008). Effect of obesity and other lifestyle factors on mortality in women with breast cancer. Int. J. Cancer.

[18] Decarli A., Franceschi S., Ferraroni M., Gnagnarella P., Parpinel M.T., La Vecchia C., Negri E., Salvini S., Falcini F., Giacosa A. (1996). Validation of a food-frequency questionnaire to asses dietary intakes in cancer studies in Italy. Results for specific nutrients. Ann. Epidemiol..

[19] Franceschi S., Negri E., Salvini S., Decarli A., Ferraroni M., Filiberti R., Giacosa A., Talamini R., Nanni O., Panarello G. (1993). Reproducibility of an Italian food frequency questionnaire for cancer studies. Results for food items. Eur. J. Cancer.

[20] Gnagnarella P., Parpinel M., Salvini S., Franceschi S., Palli D., Boyle P. (2004). The update of the Italian Food Composition Database. J. Food Comp. Anal..

[21] Trichopoulou A., Costacou T., Bamia C., Trichopoulos D. (2003). Adherence to a Mediterranean diet and survival in a Greek population. N. Engl. J. Med..

[22] Kalbfleisch J.D., Prentice R.L. (2002). The Statistical Analysis of Failure Time Data.

[23] Fine J.P., Gray R.J. (1999). A proportional hazard model for the subdistribution of a competing risk. J. Am. Stat. Assoc..

[24] Gray R.J. (1988). A class of K-sample tests for comparing the cumulative incidence of a competing risk. Ann. Stat..

[25] Dinu M., Pagliai G., Casini A., Sofi F. (2018). Mediterranean diet and multiple health outcomes: An umbrella review of meta-analyses of observational studies and randomised trials. Eur. J. Clin. Nutr..

[26] Kenfield S.A., DuPre N., Richman E.L., Stampfer M.J., Chan JM., Giovannucci E.L. (2014). Mediterranean diet and prostate cancer risk and mortality in the Health Professionals Follow-up Study. Eur. Urol..

[27] Ratjen I., Schafmayer C., di Giuseppe R., Waniek S., Plachta-Danielzik S., Koch M., Nöthlings U., Hampe J., Schlesinger S., Lieb W. (2017). Postdiagnostic Mediterranean and Healthy Nordic dietary pattern are inversely associated with all-cause mortality in long-term colorectal cancer survivors. J. Nutr..

[28] Howlader N., Cronin K.A., Kurian A.W., Andridge R. (2018). Differences in breast cancer survival by molecular subtypes in the United Staes. Cancer Epidemiol. Biomark. Prev..

[29] Kastorini C.M., Milionis H.J., Esposito K., Giugliano D., Goudevenos J.A., Panagiotakos D.B. (2011). The effect of Mediterranean diet on metabolic syndrome and its components: A meta-analysis of 50 studies and 534,906 individuals. J. Am. Coll. Cardiol..

[30] Kwon Y.J., Cho Y.E., Cho A.R., Choi W.J., Yun S., Park H., Kim H.S., Cashion A.K., Gill J., Lee H. (2020). The possible influence of Mediterranean diet on extracellular vescicle miRNA expression in breast cancer survival. Cancers.

[31] Affret A., His M., Severi G., Mancini F.R., Arveux P., Clavel-Chapelon F., Boutron-Ruault M.C., Fagherazzi G. (2018). Influence of cancer diagnosis on changes in fruit and vegetable consumption according to cancer site, stage at diagnosis and sociodemographic factors: Results from the large E3N-EPIC study. Int. J. Cancer.

[32] World Cancer Research Fund International/American Institute for Cancer Research Diet, Nutrition, Physical Activity, and Breast Cancer Survivors. Continuous Update Project Expert Report 2018..

